# Nervous and Mental Diseases

**Published:** 1902-02

**Authors:** 


NERVOUS AND MENTAL DISEASES. By ARCHIBALD CHURCH, M. D., Professor of Nervous and Mental Diseases and Head of Neurological Department, Northwestern University Medical School; and FREDERICK PETERSON, M.D., Chief of Clinic, Department of Nervous and Mental Diseases, and Clinical Lecturer on Psychiatry, College of Physicians and Surgeons, New York. Third edition, revised and enlarged. Handsome 8vo volume of 870 pages, with 322 illustrations. Philadelphia and London: W. B. Saunders & Co. 1901. [Price, cloth, $5.00 net.]

The appearance of the third edition of Church and Peterson's text-book on nervous and mental diseases within two years after the first edition was published, shows that it has been kindly accepted by the profession at large and recommended as a safe and admirable text-book. The third edition has been somewhat revised and rearranged, new tabular matter inserted, new illustrations added and otherwise improved, so that it stands today as one of the very best expositions of nervous and mental diseases in existence. The illustrations are exceptionally good, the majority of which are original and show what is expected of them. Some new ones have been incorporated, as those on adeno-lipomatosis, trophoedema and others. The part devoted to mental diseases has received careful revision by Dr. Peterson and is an admirably lucid exposition of the subject.

The book is dressed in the very best, the paper is excellent and the half-tones consequently come out to best advantage. The well known firm of Saunders & Co. are the publishers.—W.C.K.

